# Nanodroplet-mediated catheter-directed sonothrombolysis of retracted blood clots

**DOI:** 10.1038/s41378-020-00228-9

**Published:** 2021-01-06

**Authors:** Leela Goel, Huaiyu Wu, Bohua Zhang, Jinwook Kim, Paul A. Dayton, Zhen Xu, Xiaoning Jiang

**Affiliations:** 1 Department of Mechanical & Aerospace Engineering, North Carolina State University, Raleigh, NC 27695 USA; 2 The Joint Department of Biomedical Engineering, The University of North Carolina at Chapel Hill and North Carolina State University, Chapel Hill, NC 27599 USA; 3 Department of Biomedical Engineering, University of Michigan, Ann Arbor, MI 48109 USA

**Keywords:** Engineering, Nanoparticles, Biosensors

## Abstract

One major challenge in current microbubble (MB) and tissue plasminogen activator (tPA)-mediated sonothrombolysis techniques is effectively treating retracted blood clots, owing to the high density and low porosity of retracted clots. Nanodroplets (NDs) have the potential to enhance retracted clot lysis owing to their small size and ability to penetrate into retracted clots to enhance drug delivery. For the first time, we demonstrate that a sub-megahertz, forward-viewing intravascular (FVI) transducer can be used for ND-mediated sonothrombolysis, in vitro. In this study, we determined the minimum peak negative pressure to induce cavitation with low-boiling point phase change nanodroplets and clot lysis. We then compared nanodroplet mediated sonothrombolysis to MB and tPA mediate techniques. The clot lysis as a percent mass decrease in retracted clots was 9 ± 8%, 9 ± 5%, 16 ± 5%, 14 ± 9%, 17 ± 9%, 30 ± 8%, and 40 ± 9% for the control group, tPA alone, tPA + US, MB + US, MB + tPA + US, ND + US, and ND + tPA + US groups, respectively. In retracted blood clots, combined ND- and tPA-mediated sonothrombolysis was able to significantly enhance retracted clot lysis compared with traditional MB and tPA-mediated sonothrombolysis techniques. Combined nanodroplet with tPA-mediated sonothrombolysis may provide a feasible strategy for safely treating retracted clots.

## Introduction

Deep vein thromboses (DVT) affect 300,000–600,000 patients in the United States every year^1,2^. Despite the common occurrence, DVT are difficult to treat with conventional methods used for other blood clot types such as middle cerebral artery (MCA) occlusions. DVT blood clots tend to be older, denser, stiffer, and retracted as compared with unretracted clots typically encountered in MCA occlusions. Thrombolytic agent treatment such as systemic tissue plasminogen activator (tPA) administration for DVT can take over 24 hours to be effective^3–5^. However, such long treatment durations and high doses of tPA (1000 μg/ml) can greatly increase the risk of internal hemorrhage owing to off-target effects, most notably intracranial hemorrhage can lead to stroke or death^4^. Other more-targeted treatments include mechanical removal via mechanical thrombectomy. While effective at removing the clot, this technique has a risk for causing endothelial damage or producing large clot debris that lead to a pulmonary embolism^6–8^. As such, there is a need to develop site-specific DVT treatments, which minimize the tPA dose and treatment time necessary for effective clot treatment.

### Sonothrombolysis

Sonothrombolysis is a method that uses ultrasound to facilitate the removal of blood clots. Sonothrombolysis has been particularly effective at treating unretracted blood clots and improving tPA treatment by incorporating ultrasound contrast agents such as microbubbles (MBs)^9,10^. Cavitation is one of the primary mechanisms of contrast agent-mediated sonothrombolysis^9^. Stable cavitation occurs when MBs oscillate and induce localized microstreaming and acoustic streaming. Inertial cavitation occurs when the bubbles expand and rapidly collapse, inducing both micostreaming and microjets. Although stable and inertial cavitation can occur concurrently, each is active in different acoustic regimes. In addition to mechanical erosion of the clots due to cavitation, the localized streaming effects improve diffusion of thrombolytic drugs into the clot, thus improving clot lysis^11,12^.

MBs act as cavitation nuclei, which can result in stable cavitation and inertial cavitation, which are the primary mechanisms for MB-mediated sonothrombolysis. However, when it comes to applications of treating retracted clots, even MB-mediated sonothrombolysis is challenged to disrupt clots efficiently^13^. Histotripsy and high-intensity focused ultrasound (HIFU) have also been used in pre-clinical studies to treat retracted clots, however, these methods use high peak negative pressures (PNPs) of up to 15 MPa, which requires a large external ultrasound transducer, thus these techniques cannot be used to treat vessel locations with ultrasound blockage, e.g., pulmonary embolism with vessels blocked by the lung or ribs^14–19^.

### Nanodroplet-mediated sonothrombolysis

Nanodroplets (NDs) are an ultrasound contrast agent, which has recently been applied not only for enhanced ultrasound imaging, but for enhanced drug delivery and more recently, sonothrombolysis of retracted clots^20–24^. Upon insonation with sufficient PNPs, nanodroplets are able to transition from liquid droplets to gas-filled bubbles, which then have similar acoustic properties and behaviors as MBs. Given the small size of NDs (100–300 nm) compared with MBs (1–10 μm), NDs have the potential to penetrate into retracted clots with low porosity. Upon insonation, these droplets can then form microchannels within the clot and act to both mechanically break down the clot via cavitation effects and allow for greater diffusion of tPA and cavitation effects inside the clots^20,25^.

For ND-mediated sonothrombolysis, typical studies use an external HIFU device for these excitations in order to activate the droplets^22–24^. Although initial studies are promising, it requires the PNPs of 3–5 MPa to activate the ND, which exceeds the safety limit for ultrasound imaging and causes concerns for clinical translation. Low-boiling point phase change NDs may overcome the limitations of current ND techniques by reducing the pressure necessary for ND activation and by extension clot lysis, thus allowing a lower PNP output necessary to treat retracted clots^22,25,26^. These low-boiling point phase change nanodroplets are designed to be stable at up to 37^∘^ C and have a vaporization threshold at ~1 MPa^26^.

### FVI transducer for sonothrombolysis

**Fig. 1:**
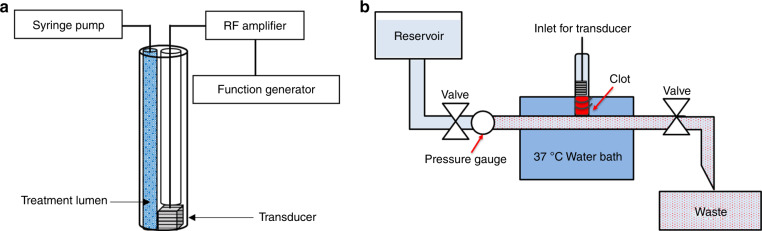
Experimental setup. Diagram of **a** forward-viewing intravascular transducer catheter assembly and **b** experimental flow model

Our group has developed a forward-viewing intravascular (FVI) sub-megahertz transducer for sonothrombolysis, which is effective for tPA and MB-mediated sonothrombolysis in unretracted clot models (Fig. 1a)^27–29^. An intravascular system does not have the treatment location limitation of using an external transducer. In addition, this transducer allows for site-specific insonation and treatments of clots, and may limit unintended off target effects given that ultrasound contrast agents, thrombolytic drugs, or potentially magnetic MBs are directly injected onto the clot surface. Compared with a clinical standard side viewing intravascular transducer that can only treat partially blocked vessels, this FVI system enables treatment of both partially and completely blocked vessels^30^. Previous studies from our group have determined optimal transducer excitation parameters for MB-mediated sonothrombolysis for unretracted clots^27^. We then were able to combine MBs with low dose of tPA to further enhance clot lysis outcomes in unretracted clots, and further understand the synergistic effects of combining tPA with MBs when using our FVI sonothrombolysis transducer^29^.

In this paper, we present the first application of our FVI transducer to treat retracted clots. Moreover, this is the first demonstration of the use of nanodroplets with intravascular ultrasound for sonothrombolysis treatments.

### Purpose

Given the potential for ND-mediated sonothrombolysis for site-specific treatment of retracted clots via targeted injection from our FVI transducer system, in this study, we investigated the feasibility of using our FVI transducer combined with ND and tPA for sonothrombolysis in retracted clots. First, we evaluated the ability of the FVI transducer to activate low-boiling point phase change nanodroplets and generate cavitation via passive cavitation detection (PCD). We then demonstrated that ND-mediated sonothrombolysis, in combination with tPA can treat retracted clots.

## Results

### Nanodroplet activation

**Fig. 2:**
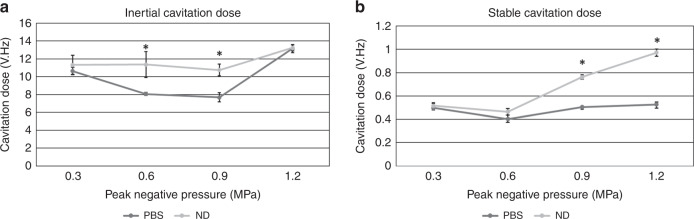
Nanodroplet activation. **a** Inertial and **b** stable cavitation doses for nanodroplet mediated (ND) sonothrombolysis with different peak negative pressures compared with a control group of phosphate buffered saline (PBS) with ultrasound. The ultrasound excitation parameters were 700kHz, 10ms pulse length, and 20 cycles. * indicates *p* < 0.05 compared with the control group (*N* = 3)

The stable cavitation dose increased with increasing PNP output and were 0.52 ± 0.02*V* ⋅ *Hz*, 0.46 ± 0.03*V* ⋅ *Hz*, 0.76 ± 0.02*V* ⋅ *Hz*, 0.97 ± 0.03*V* ⋅ *Hz* for 0.3 MPa, 0.6 MPa, 0.9 MPa, and 1.2 MPa, respectively (Fig. 2b). There was a statistically significantly increased stable cavitation dose at 0.9 MPa and 1.2 MPa compared with untreated controls (*p* < 0.05) and approached significance at 0.6 MPa (*p* = 0.057). 0.3 MPa did not generate increased stable cavitation compared with the control group. The inertial cavitation dose increased with increasing PNP output and were 11.33 ± 1.07*V* ⋅ *Hz*, 11.38 ± 1.44*V* ⋅ *Hz*, 10.77 ± 0.67*V* ⋅ *Hz*, 13.22 ± 0.35*V* ⋅ *Hz* for 0.3 MPa, 0.6 MPa, 0.9 MPa, and 1.2 MPa, respectively (Fig. 2a). For the inertial cavitation doses with there was a statistically significantly increased dose at 0.6 MPa and 0.9 MPa compared with untreated controls (*p* < 0.05). These results indicate that the primary mechanism is in the stable cavitation regime.

### Retracted clot lysis

**Fig. 3:**
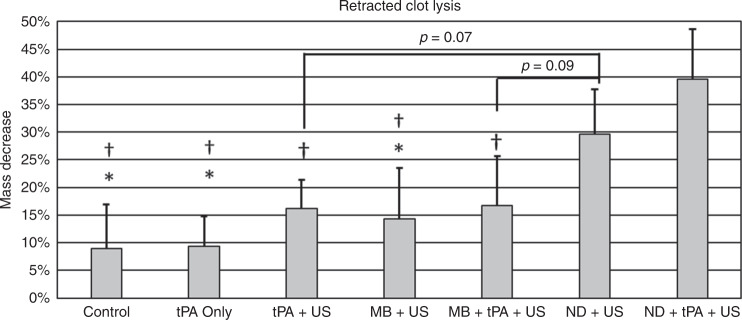
Retracted clot lysis results for different treatment conditions with a peak negative pressure of 0.9 MPa. * indicates *p* < 0.05 compared with ND+US and ^†^ indicates *p* < 0.05 compared with ND+tPA+US. (*N* = 6)

The percent mass decrease in retracted clots was 9 ± 8%, 9 ± 5%, 16 ± 5%, 14 ± 9%, 17 ± 9%, 30 ± 8%, 40 ± 9% for the control group, tPA alone, tPA + US, MB + US, MB + tPA + US, ND + US, and ND + tPA + US groups, respectively. ND + tPA-mediated sonothrombolysis outperformed the tPA alone, tPA + US, MB-mediated sonothrombolysis, and MB + tPA-mediated sonothrombolysis (Fig. 3). The ND + US group statistically outperformed the control, tPA only, and MB + US groups and approached significance for the tPA + US (*p* = 0.07) and MB + tPA + US (*p* = 0.09) groups. In addition, ND and ND + tPA-mediated sonothrombolysis were the only conditions, which statistically improved retracted clot lysis compared with the control group. A similar comparison study was conducted in unretracted clots, where it was shown that ND-mediated sonothrombolysis performs as well as tPA mediated thrombolysis and MB-mediated sonothrombolysis, and agrees well with our previous study on microbubble and tPA-mediated clot lysis in unretracted clots (S2). One-way analysis of variance and Tukey’s honest squared difference was used to assess significance between treatment groups for the thrombolysis experiments. The significance level was set to *p* < 0.05.

## Discussion

We demonstrated the first use of ND + tPA-mediated sonothrombolysis with a FVI transducer for effective lysis of retracted clots. This technique achieved effective treatment of retracted clots without the need for high amplitude ultrasound treatment such as with high-intensity focused ultrasound or histotripsy. In unretracted clots, this technique is as effective as tPA-mediated sonothrombolysis and MB-mediated sonothrombolysis. Our results indicate that these low-boiling point NDs may be an alternative to traditional thrombolytic approaches to improve the treatment of retracted clots while decreasing the potential negative side-effects of thrombolytic treatments.

We also demonstrated that these NDs, when combined with tPA, can significantly enhance retracted clot lysis compared with MB and MB + tPA mediated approaches. Our results correspond well with a study by Sutton et al.^13^ comparing MB and tPA-mediated sonothrombolysis in unretracted and retracted clots, wherein MB-mediated sonothrombolysis did not improve retracted clot lysis. Here, we demonstrate that NDs can be used to enhance tPA-mediated sonothrombolysis in retracted clots. In addition, this was achieved using a relatively low PNP of 0.9 MPa (within the FDA safety limit mechanical index = 1.9) compared with higher PNP’s of up to 15 MPa as used in other external sonothrombolysis techniques for retracted clots^18^. Previous studies utilizing nanodroplets using high intensity focused ultrasound with PNPs ranging from 2 to 8 MPa were conducted using fresh, unretracted clots, and resulted in percent mass decreases of 25–80% after 30 minutes of treatment^22,24,25^. In this study, we were able to achieve a percent mass decrease of 40% using only 0.9 MPa under similar conditions in retracted clots. This demonstrates that low-boiling point, phase change nanodroplets may be a useful technique in overcoming the limitations of retracted clot treatment, whereas minimizing the acoustic energy needed for sonothrombolysis. In addition, current HIFU sonothrombolysis techniques are subcutaneous, and the insonation has to travel through different tissue layers before reaching the clot of interest. In our intravascular technique, we are able to directly insonate the clot of interest, thus allowing all of the acoustic energy to be directed to the clot.

Although exciting, future work should involve assessing the safety of ND-mediated sonothrombolysis. Metrics such as clot debris size and vessel damage should be assessed in order to understand the safety of ND-mediated sonothrombolysis with the FVI transducer compared with HIFU techniques for retracted clots. The parameters used in this study were based on our previous MB-mediated results. One limitation of our study is the use of an in vitro venous flow model, which may not fully replicate in vivo human conditions. Further study may be done to improve the in vitro flow model and optimize the internal flow conditions to understand its influence on thrombolytic outcomes and more accurately model venous flow. Future parameter optimization, such as duty cycle, treatment duration, and pulse width, is also warranted to increase the treatment efficacy for nanodroplet-mediated sonothrombolysis. Cavitation was detected and assumed to be the mechanism of ND-mediated sonothrombolysis. In addition to cavitation, we hypothesize that the small size of the nanodroplets allows them to penetrate into the dense fibrin clot before activation, thus forming larger channels for the tPA to diffuse into the clot and improve efficacy. Further mechanistic studies are warranted for improvement of ND-mediated sonothrombolysis.

We demonstrated the use of low-boiling point phase change nanodroplets for intravascular sonothrombolysis. In addition, we showed that combining ND + tPA + US can significantly improve retracted clot lysis, which may improve current approaches for treating retracted clots. Our FVI transducer can be used for nanodroplet-mediated sonothrombolysis to treat both retracted and unretracted clots using a low PNP compared with other high-intensity sonothrombolysis techniques.

## Materials and methods

### FVI transducer

The FVI transducer was fabricated similarly to previous studies^27,29^. The transducer was composed of seven stacked layers of PZT-5A, E-solder 3022 bonding layers, and an alumina epoxy matching layer. The final aperture size was 1.5 mm. The center frequency of the transducer was 700 kHz with a bandwidth of 445 kHz–1.05 MHz, the impedance of 225 Ω, and capacitance of 1.43 nF. The peak to peak driving voltages of the transducer were 15 V, 35V , 50 V, and 70 V to achieve PNP outputs of 0.3 MPa, 0.6 MPa, 0.9 MPa, and 1.2 MPa, respectively, corresponding to a sensitivity of 0.02 MPa/V and driving power of 11 W.

### PCD for nanodroplet activation

The minimum PNP necessary for ND activation was determined using PCD, performed similarly to our previous study^29^. The PNP of the FVI transducer was varied from 0.3 MPa to 1.2 MPa. Nanodroplets (10^8^ ND/ml) or phosphate-buffered saline (PBS) were injected via the integrated catheter of the transducer at a rate 0.1 ml/min via a syringe pump. The transducer excitation parameters were a center frequency of 700 kHz, 10 ms pulse length, and 20 cycles. Cavitation signals were detected using a hydrophone (HGL-0085, ONDA Corp., Sunnyvale, CA, USA). Three radio frequency (RF) signals were collected for each condition. Inertial and stable cavitation doses were calculated using MATLAB (MATLAB R2018b, Mathworks, Natick, MA, USA). The stable cavitation dose was calculated based on the area under the curve of the second harmonic (2*f*0 ± 0.5*f*0) of the frequency spectrum of the RF data. The inertial cavitation dose was calculated based on the area under the curve of the broadband noise of the 3rd–6th harmonics, wherein the fundamental harmonics (3*f*0 ± 0.2*f*0, 4*f*0 ± 0.2*f*0, 5*f*0 ± 0.2*f*0, 6*f*0 ± 0.2*f*0) were subtracted from the whole AUC in this range. From here, it was determined that a PNP of 0.9 MPa was sufficient for ND activation, which matches well with previous studies on the vaporization pressure necessary for these droplets^26^.

### Clot lysis

Upon demonstrating the feasibility of ND-mediated sonothrombolysis in retracted and unretracted clots (S1), we sought to further enhance retracted clot lysis with NDs and to compare ND-mediated sonothrombolysis to existing sonothrombolysis techniques. In order to enhance retracted clot lysis, we combined NDs with a low dose of tPA. Given the success of MB mediated sonothrombolysis with a low-dose tPA in unretracted clots, we chose to combine ND-mediated sonothrombolysis with a low dose tPA^29^. We compared ND + tPA-mediated sonothrombolysis to a control of PBS alone without ultrasound, tPA treatment alone without ultrasound, tPA with ultrasound, ND-mediated thrombolysis, MB mediated sonothrombolysis, and MB + tPA-mediated sonothrombolysis.

#### Clot preparation

Bovine whole blood was used to prepare clots as done previously^18,27–29,31–35^. Acid citrate dextrose anticoagulated blood was mixed with 2.75% Calcium Chloride in a 10:1 ratio (50 mL blood/5 mL CaCl_2_). Unretracted clots were prepared by adding the blood mixture to plastic centrifuge tubes. Retracted clots were prepared by adding the blood mixture to borosilicate glass pipettes as described by Sutton et al.^13,18^. Clots were then incubated in a 37 °C water bath for 3 hours. The clots were then stored in a refrigerator at 4 °C for 2–16 days. For experiments, the retracted and unretracted clots were prepared to a final length of 10 mm, diameter of 5 mm, final mass of 150 ± 20 mg. Clot characterization results can be found in the supplementary results (S3). A total of 54 retracted clots and 33 unretracted clots were tested in this study.

#### Treatment condition preparation

Lipid-shelled, decafluorobutane (DFB) gas core nanodroplets were prepared as previously described^25,26^. In brief, lipid-shelled DFB MB were synthesized and condensed into liquid-core nanodroplets with an average size of 100–200 nm and concentration of 10^10^ ND/ml. Nanodroplets were then diluted with PBS to a final concentration of 10^8^ ND/ml for all treatment conditions. The microbubbles themselves have a mean microbubble diameter of 1.1 μm, with an initial concentration of 10^10^ MB/ml. In the MB treatment conditions, the final concentration was 10^8^ MB/ml.

TPA (Cathflo Activase, Genentech USA, Inc) was prepared via the manufacturer’s instructions to a concentration of 1000 μg/ml, aliquoted, and stored at −20 °C until use. For experiments, tPA was then diluted to the working concentration of 1.0 μg/ml using PBS. In the combined ND + tPA and MB + tPA conditions, tPA was added to the diluted ND and MB solutions to a final concentration of 1.0 μg/ml.

#### Experimental setup

Experiments were performed in an in vitro DVT venous flow model, similar to the apparatus used by Zhang et al.^34^. The flow model consisted of a custom acrylic reservoir of degassed water, 37 °C water bath (Poly ProBath, Revolutionary Science, Shafer, MN, USA), and waste container (Fig. 1b). The pressure was maintained at 3.5 ± 0.5 mmHg and monitored with a digital pressure gauge (DPGA-04, Dwyer Instruments, Inc., Michigan City, IN, USA). The height of the reservoir and one-way valves were used to control the one-way flow and pressure of the water through the system. The flow rate was 50 mL/min. An inlet channel with a mesh netting was used to place the clot sample. The mesh netting prevented the clot from flowing through the system and maintain a partial occlusion in the tubing. Once the clot was placed, the FVI transducer with integrated treatment catheter was placed through the same inlet in order to treat the clot.

The FVI transducer was installed into an integrated catheter system (Sonovascular, Inc.) with a lumen for the treatment injection. The total catheter diameter is 3 mm. The transducer face was maintained to be <1 mm away from the clot surface throughout the treatment. The center frequency of the transducer was 700 kHz with a duty cycle of 5.7% (200 cycles, 5 ms pulse length). As implemented in our previous studies, in order to ensure penetration of ultrasound contrast agents into the clot, the ultrasound output was manually switched “on” for 2 minutes (5.7% duty cycle) and “off” for 30 seconds throughout the 30-minute treatment period to allow for penetration of the treatment solutions into the clot^27,29^.

The thrombolysis experiments compared various sonothrombolysis techniques to nanodroplet-mediated sonothrombolysis techniques. tPA was prepared as described previously^29^. The treatment conditions were PBS alone with no ultrasound (control), tPA alone with no ultrasound (1.0 μg/ml), tPA + US, MB + US (10^8^ MB/ml), MB + tPA + US (10^8^ MB/ml, 1.0 μg/ml), ND + US (10^8^ ND/ml, 1.0 μg/ml), and ND + tPA + US. The PNP used for these experiments was 0.9 MPa. Similar to the feasibility experiment, each treatment was injected via the integrated catheter directly onto the clot surface at a rate 0.1 ml/min for 30 minutes via a syringe pump. Each condition was tested six times. The metric for clot lysis was the percent mass decrease of the clot before and after each treatment.

## Supplementary information

Supplementary material
