# Supplementary material for: Nanodroplet-mediated catheter-directed sonothrombolysis of retracted blood clots
Source: Microsyst Nanoeng. 2021 Jan 6;7:3. doi: 10.1038/s41378-020-00228-9 (PMC7787976; doi:10.1038/s41378-020-00228-9)
Supplement: Supplementary file 1 — Supplementary material [file 41378_2020_228_MOESM1_ESM.docx]

**Nanodroplet Mediated Catheter-Directed Sonothrombolysis of Retracted Blood Clots**

Leela Goel,^1, 2^ Huaiyu Wu,^1^ Bohua Zhang,^1^ Jinwook Kim,^2^ Paul A. Dayton,^2^ Zhen Xu,^3^ and Xiaoning Jiang^1,*^

1) Department of Mechanical & Aerospace Engineering, North Carolina State University, Raleigh, NC, 27695, USA

2) The Joint Department of Biomedical Engineering, The University of North Carolina at Chapel Hill and North Carolina State University, Chapel Hill, NC, 27599, USA

3) Department of Biomedical Engineering, University of Michigan, Ann Arbor, MI, 48109, USA

*Corresponding author: Xiaoning Jiang

Address: 3282 Engineering Building III, 911 Oval Dr. Raleigh, NC, 27606, United States

E-mail: xjiang5@ncsu.edu

Phone: 919-515-5240

**Supplementary Figures**

**
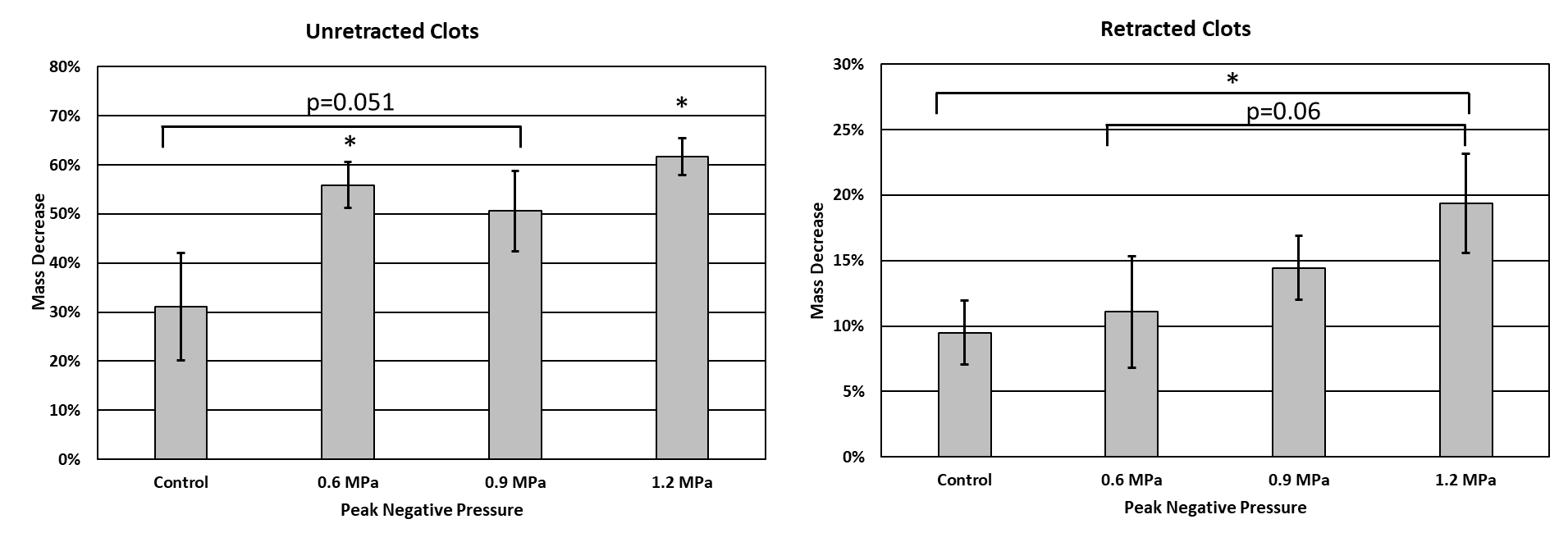
**

**Supplementary 1.** Percent mass decrease of a) unretracted and b) retracted clots for nanodroplet mediated sonothrombolysis at different peak negative pressures. * indicates p<0.05 compared to the control group in each case.


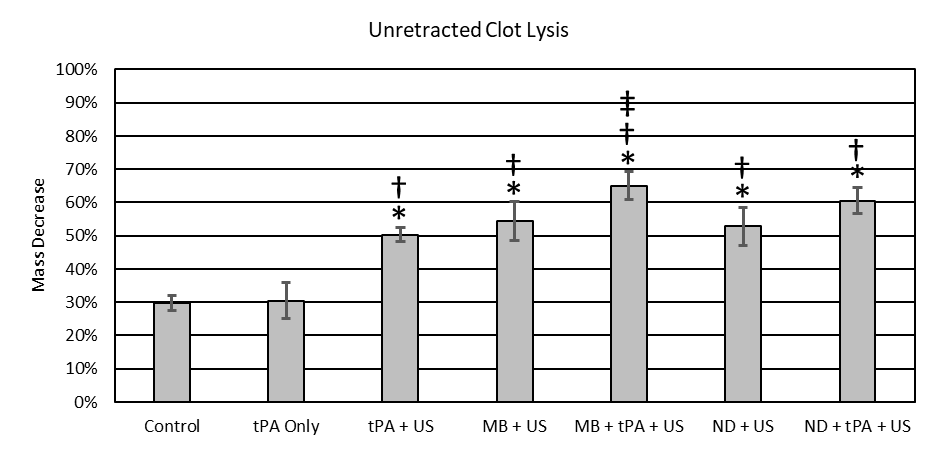


**Supplementary 2.** Unretracted clot lysis results for different treatment conditions with a peak negative pressure of 0.9 MPa. * indicates p<0.05 compared to control, † indicates p<0.05 compared to tPA only, and ‡ indicates p<0.05 compared to tPA + US.


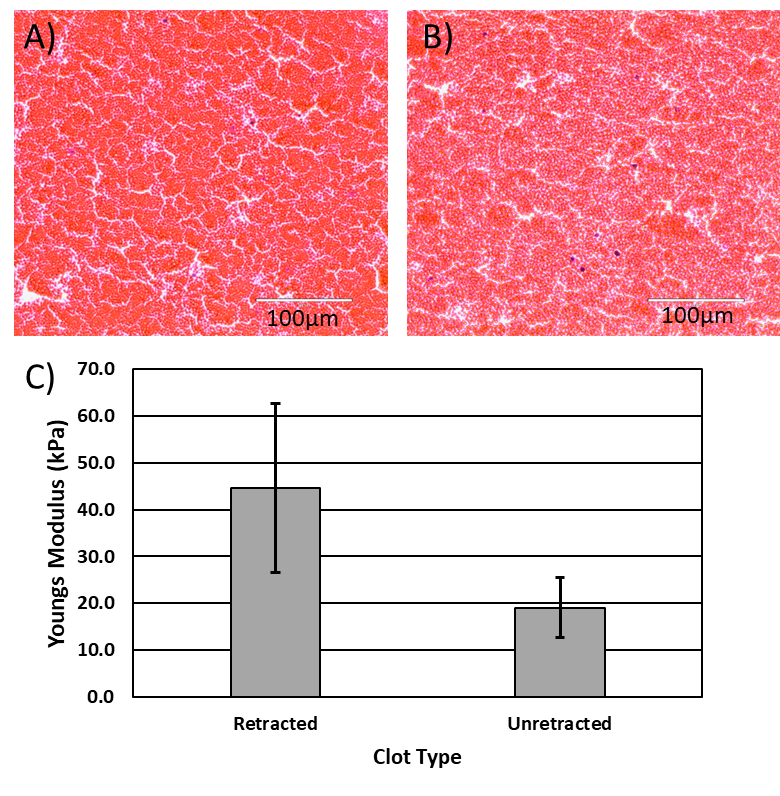


**Supplementary 3.** Verification of retracted and unretracted clots. Histology slides of A) retracted clot and B) unretracted clot. C) Calculated Young's modulus of clots.

The degree of clot retraction was then verified using histology and stiffness measurements. Hemoxatocin and Eosin (H&E) staining was conducted on the retracted and unretracted clot samples. Samples were prepared to 5µm thickness and imaged using an EVOS compound light microscope (EVOS® FL Auto Imaging System, Life Technologies Corporation, Carlsbad, CA, USA). The porosity of the clots was calculated via the percent of white pixels in the images using a custom image processing algorithm (MATLAB R2018b, Mathworks, Natick, MA, USA). Clot stiffness was characterized via uniaxial testing. Clots were mounted to a BioTester mechanical testing system (BioTester, Cell Scale, Waterloo, ON, Canada). The specimens were then subject to cyclical loading under 3 strain conditions. Young’s modulus was calculated from the resulting force-displacement curves, providing comparative stiffness values for retracted and unretracted clots.
